# Diagnostic performance of a colorimetric RT -LAMP for the identification of SARS-CoV-2: A multicenter prospective clinical evaluation in sub-Saharan Africa

**DOI:** 10.1016/j.eclinm.2021.101101

**Published:** 2021-08-28

**Authors:** Marycelin Mandu Baba, Molalegne Bitew, Joseph Fokam, Eric Agola Lelo, Ahmed Ahidjo, Kominist Asmamaw, Grace Angong Beloumou, Wallace Dimbuson Bulimo, Emanuele Buratti, Collins Chenwi, Hailu Dadi, Pierlanfranco D'Agaro, Laura De Conti, Nadine Fainguem, Galadima Gadzama, Paolo Maiuri, Janet Majanja, Wadegu Meshack, Alexis Ndjolo, Celine Nkenfou, Bamidele Soji Oderinde, Silvanos Mukunzi Opanda, Ludovica Segat, Cristiana Stuani, Samwel L. Symekher, Desire Takou, Kassahun Tesfaye, Gianluca Triolo, Keyru Tuki, Serena Zacchigna, Alessandro Marcello

**Affiliations:** a Department of Medical Laboratory Science, College of Medical Sciences, University of Maiduguri, Borno State P.M.B.1069, Nigeria; b Ethiopian Biotechnology Institute (EBTI), Addis Ababa, Ethiopia; c The Chantal Biya International Reference Center (CIRCB), Yaounde, Cameroon; d Department of Medical Laboratory Science, Faculty of Health Science, University of Buea, Buea, Cameroon; e Center for Biotechnology Research, Kenya Medical Research Institute (KEMRI), Nairobi, Kenya; f International Center for Genetic Engineering and Biotechnology (ICGEB), Padriciano 99, Trieste 34149, Italy; g Azienda Sanitaria Universitaria Integrata di Trieste, UCO Igiene e Sanità Pubblica, Trieste, Italy; h Dipartimento di Scienze Mediche Chirurgiche e della Salute, Università di Trieste, Italy; i IFOM, the FIRC Institute of Molecular Oncology, Via Adamello 16, Milano 20139, Italy

**Keywords:** SARS-CoV-2, COVID-19, coronavirus, reverse transcription loop-mediated isothermal amplification, RT LAMP, multicentre, prospective, Observational study, diagnostic accuracy, Sub-Saharan Africa, Cameroon, Ethiopia, Kenya, Nigeria, Italy

## Abstract

**Background:**

Management and control of the COVID-19 pandemic caused by the severe acute respiratory syndrome coronavirus SARS-CoV-2 is critically dependent on quick and reliable identification of the virus in clinical specimens. Detection of viral RNA by a colorimetric reverse transcription loop-mediated isothermal amplification (RT-LAMP) is a simple, reliable and cost-effective assay, deployable in resource-limited settings (RLS). Our objective was to evaluate the intrinsic and extrinsic performances of RT-LAMP in RLS.

**Methods:**

This is a multicenter prospective observational study of diagnostic accuracy, conducted from October 2020 to February 2021 in four African Countries: Cameroon, Ethiopia, Kenya and Nigeria; and in Italy. We enroled 1657 individuals who were either COVID-19 suspect cases, or asymptomatic and presented for screening. RNA extracted from pharyngeal swabs was tested in parallel by a colorimetric RT-LAMP and by a standard real time polymerase chain reaction (RT-PCR).

**Findings:**

The sensitivity and specificity of index RT LAMP compared to standard RT-PCR on 1657 prospective specimens from infected individuals was determined. For a subset of 1292 specimens, which underwent exactly the same procedures in different countries, we obtained very high specificity (98%) and positive predictive value (PPV = 99%), while the sensitivity was 87%, with a negative predictive value NPV = 70%, Stratification of RT-PCR data showed superior sensitivity achieved with an RT-PCR cycle threshold (Ct) below 35 (97%), which decreased to 60% above 35.

**Interpretation:**

In this field trial, RT-LAMP appears to be a reliable assay, comparable to RT-PCR, particularly with medium-high viral loads (Ct < 35). Hence, RT-LAMP can be deployed in RLS for timely management and prevention of COVID-19, without compromising the quality of output.

**Funding:**

Bill & Melinda Gates Foundation [Grant No. INV-022,816].

**Research in context**

**Evidence before this study**

Colorimetric reverse transcription loop-mediated isothermal amplification (RT-LAMP) of SARS-CoV-2 genomes is a nucleic acid test alternative to gold-standard RT-PCR. This technology attracted a lot of attention as a diagnostic tool, particularly for deployment in resource-limited settings. However, large multicenter clinical trials of diagnostic accuracy in Africa have not been conducted so far to assess its potential.

**Added value of this study**

The study was conducted independently in Cameroon, Ethiopia, Kenya, Nigeria and in Italy providing a first evaluation of the efficacy of the technology in low- and middle-income countries and least developed countries in sub-Saharan Africa. Results are therefore significant for the deployment of RT-LAMP as a viable alternative to RT-PCR for diagnostic and surveillance.

**Implications of all the available evidence**

While RT-PCR remains the most sensitive diagnostic tool for SARS-CoV-2 detection, colorimetric RT-LAMP showed a very good specificity and a comparable sensitivity for medium-high viral loads, which are the most relevant for virus transmission. RT-LAMP is therefore a cost-effective and easy to use diagnostic test that can be implemented for resource-limited settings while maintaining a high quality of response.

Alt-text: Unlabelled box

## 1 Introduction

The coronavirus disease 2019 (COVID-19) pandemic, caused by the severe acute respiratory syndrome coronavirus-2 (SARS-CoV-2), is a global threat that entails tremendous opportunities to accelerate research and development, with implications beyond this crisis that could further impact the management of infectious diseases. It is critical that new developments are designed to minimise the technological gap between countries, as a pandemic needs to be managed locally in order to achieve global control.

Diagnosis of SARS-CoV-2 currently relies extensively on the real time polymerase chain reaction (RT-PCR), a robust technology with high sensitivity and specificity [1], [2], [3], [4]. Even though the mass testing of asymptomatic individuals is of paramount importance in breaking the transmission chain, using RT-PCR entails a heavy workload on health care, until vaccines are rolled out effectively. Molecular tests have an advantage over antigen detection tests, not only for their superior specificity and sensitivity, but also for their quicker adaptability to emerging variants. However, although RT-PCR use is widespread, its accessibility is limited in certain areas due not only to the cost of equipment, but also to the scarcity of reagents in times of high demand and the need for highly trained personnel. Such logistic and technical challenges are particularly frequent in low- and middle-income countries (LMICs) and least developed countries (LDCs) in sub-Saharan Africa (SSA) [5], which contain 17.2% of the world's population and yet have reported to date only around 3% (4 million) of the global COVID-19 infections (∼130 million). The most recent figures from the coronavirus epidemic [6], clearly show that in SSA, Cameroon, with a population of 27.2 Million (M) has performed ∼64,200 PCR tests/M and reported ∼3000 cases/M; Ethiopia (population 118 M) has tested ∼25,.600/M and reported ∼2300 cases/M; Kenya (population 55 M) has tested ∼38,800/M and reported ∼3700 cases/M; while Nigeria (population 211 M) has tested ∼11,500 /M and reported 824 cases/M. In comparison in Europe, Italy (population 60 M) has tested ∼1280,000/M and reported ∼72,150 cases/M. These data clearly demonstrate that testing capacity for COVID-19 remains critically low in SSA and cost-effective alternatives must be carefully considered.

An alternative molecular test to RT-PCR is the reverse transcription loop-mediated isothermal amplification (RT-LAMP) [7]. In a nutshell, RT-LAMP requires a reverse transcriptase, a thermostable DNA polymerase with strong strand displacement activity, and up to six DNA oligonucleotides. The reaction is frequently conducted at a constant temperature of 65 °C for 30 min and results in the amplification of reverse-transcribed DNA from a specific template. RT-LAMP is a cost-effective diagnostic test also because the cost per reaction, calculated in this study, is roughly one-third of that for RT-PCR. Furthermore, the RT-LAMP assay does not require expensive equipment, such as a thermal cycler with real-time fluorescence measurement, because readout of the RT-LAMP can be simply colorimetric, with a pH dependent shift of colour for a positive reaction [8]. Also the step of RNA extraction, common to both RT-PCR and RT-LAMP, could be skipped both from swabs or directly from saliva with minimal loss of sensitivity and a great advantage in terms of savings as several reports have been proposing [[9], [10], [11], [12], [13], [14]]. Colorimetric RT-LAMP represents a formidable alternative to RT-PCR, but comprehensive clinical studies are still required for evidence-based decision-making towards its broad implementation across geographical settings. Such findings would help in closing the gaps in SARS-CoV-2 diagnosis in RLS, and would also accelerate result delivery due to the short turn-around-time and the user-friendly procedure of the RT-LAMP assay. With the goal of contributing to the accuracy in detecting SARS-CoV-2 in RLS, we sought to evaluate the diagnostic performance of RT-LAMP (index test) in terms of intrinsic (sensitivity, specificity) and extrinsic (positive and negative predictive values) characteristics, according to SARS-CoV-2 viral load estimates provided by the conventional RT-PCR (reference test) in LMIC/LDC of SSA.

## 2 Materials and methods

### 2.1 Study design

This is a multicenter, observational and cross-sectional study on swab samples collected prospectively from 1657 individuals who referred for SARS-CoV-2 molecular testing in four African Countries and in Italy. In certain very limited cases, due to shortage of fresh samples, previously-stored swabs were used and re-tested in parallel with both RT-PCR and RT-LAMP assays, to avoid biases due to storage conditions. Ethical approval has been granted by governing bodies in each participating country and all individuals provided written informed consent.

Participating institutions were the following:

Cameroon (CMR): The Chantal Biya International Reference Centre (CIRCB), Yaounde, is a national reference laboratory for SARS-CoV-2 testing. Samples were collected at two locations: Palais de Sports and Ecole de Police, then analysed and tested at CIRCB. Ethical approval for this study number CNERSH 1101/15/2020.
Ethiopia (ETH): The Ethiopian Biotech Institute (EBTI) is the national reference laboratory for the testing and diagnosis of SARS-CoV-2. Samples were provided through the Ethiopian Public Health Research Institute from four reference hospitals, Kadisco General Hospital, Myungsung Christian Medical center (MCM), International Cardiovascular Medical Centre Hospital (ICMC), and Saint Yared General Hospital. Ethical approval for this study number EBTI/001/2021.
Italy (ITA): The Azienda Sanitaria Universitaria Giuliano Isontina (ASUGI) of Trieste was the clinical Centre that collected the samples from patients and performed the reference diagnostic testing; while the International Centre for Genetic Engineering and Biotechnology (ICGEB) Trieste was the laboratory for the analysis and testing of the samples for this study. Ethical approval for this study number 103_2020H.Kenya (KEN): The Centre for Virus Research and Centre for Biotechnology Research, Kenya Medical Research Institute (KEMRI) was the national laboratory responsible for the collecting, testing and diagnosis of SARS-CoV-2. Ethical approval for this study number KEMRI/SERU/CBRD/218/4131.
Nigeria (NIG): The COVID-19 Laboratory, hosted in the WHO National Polio Laboratory, University of Maiduguri Teaching Hospital, Maiduguri, Borno State. The laboratory was accredited by the Nigerian center for Disease Control (NCDC) for the diagnosis of SARS-CoV-2 in Borno State, Northern-Eastern Nigeria. Ethical approval for this study number UMTH/REC/659.

Pharyngeal swab samples were collected between October 2020 and February 2021 from individuals who had been referred to the indicated participating centres for SARS-CoV-2 diagnosis. Swab samples to be included in the current study were selected according to the result of the reference RT-PCR test at the diagnostic laboratory in each Country with the aim of including 25% negative samples, 50% positive samples (i.e., Ct<30) and 25% weakly positive samples (i.e., Ct≥30) for each centre. RT-PCR and RT-LAMP tests were made freely available to each participating centre. The primary endpoint considered was the sensitivity of the RT-LAMP test (index test) compared with the Liferiver RT-PCR (reference test). The population for this endpoint consisted of samples positive for SARS-CoV-2 at the RT-PCR test with Ct<30, and samples negative by the RT-PCR test (eligibility criteria). Test sensitivity was also calculated for the subgroup of swabs with Ct≥30 (low viral load, secondary end-point of the study). Other variables collected included: gender, age, hospitalization, clinical diagnosis (if available), symptoms and severity, onset of symptom date (if available), specimen collection date, specimen type (upper/lower respiratory tract), type of RT-PCR assay.

Data access at the country level was the following: Marycelin M Baba (Nigeria), Pierlanfranco D'Agaro (Italy), Molalegne Bitew (Ethiopia), Joseph Fokam (Cameroon) and Eric A Lelo (Kenya). Access to the complete data set: Laura De Conti, Paolo Maiuri and Alessandro Marcello.

### 2.2 Procedures

Samples were processed for diagnostic purposes according to the standard RT-PCR procedure in place at each participating centre. Swab collection and centralized laboratory testing were performed by separate staff members. Immediately after the RT-PCR results were made available (<48 h), the same samples were re-extracted with the QIAamp viral RNA mini kit (Qiagen #52,906). Extracted RNA was tested by RT-PCR with the novel coronavirus (2019-nCoV) real-time multiplex RT-PCR kit (Liferiver #RR-0479-02, Revision No. ZJ0010 produced in China) [15,16] and by the SARS-CoV-2 rapid colorimetric LAMP assay kit (New England Biolabs #E2019S produced in US) [17]. The Liferiver RT-PCR targets three gene loci separately in the SARS-CoV-2 genome: Orf1ab, E and N, while the RT-LAMP from New England Biolabs targets *E* and *N* simultaneously. Conservative criteria for positivity of the RT-PCR were at least two targets out of three, while criteria for positivity for the RT-LAMP was colour change from pink to yellow after 30 min of incubation at 65 °C, according to product specifications. Negative samples with negative internal controls and very rare samples with poor development of the colour reaction were excluded from the analysis. All countries performed the same procedures according to manufacturers’ instructions and shared standard protocols, with the exception of Cameroon who proceeded only to RT LAMP on RNA extracted with the QIAamp viral RNA mini kit (Qiagen #52,906) while RT-PCR was performed with the Abbott RealTime SARS-CoV-2 assay (Abbott Laboratories, USA), as per the manufacturer's instructions. For this reason, the data from Cameroon were treated separately form the others when compiling the stratification analysis where all data from the other countries, which were undergoing the same procedures, were considered as a whole.

Quantification of SARS-CoV-2 genomes was performed using a synthetic viral RNA as previously described with a procedure and reagents freely available through the ICGEB [18,19]. Calculated logarithmic dilutions of viral RNA from a laboratory isolate (SARS-CoV-2 ICGEB-FVG_5) [20] were plotted against average Ct values of the Liferiver RT-PCR method from two independent replicates in triplicate repeats to obtain a non-linear regression standard curve where to interpolate unknown values using GraphPad Prism version 9.0.

### 2.3 Statistical analysis

The confusion matrix (contingency 2 × 2 table) method for data analysis was used to derive parameters such as: sensitivity, specificity, negative and positive predictive values, precision and accuracy of the index assay compared with the reference. The Cohen's Kappa coefficient was used to measure inter-rater reliability. Data were analysed with R [21]. To compare the assay with the reference, the “caret” package [https://CRAN.R-project.org/package=caret] was used to compute the main confusion matrix statistics. The “irr” package [https://CRAN.R-project.org/package=irr] was used to compute the value of the Cohen's Kappa, a parameter commonly used to measure inter-rater reliability [22]⁠, while the “vcd” package [https://CRAN.R-project.org/package=vcd] was employed to obtain its 95% confidence interval. For data refining, assay sensitivity and accuracy were calculated considering only reference positive cases in function of their RT-PCR results. Results were finally visualized using “ggplot2” [https://CRAN.R-project.org/package=ggplot2] and “gridExtra” [https://CRAN.R-project.org/package=gridExtra] packages.

### 2.4 Role of funding source

Funds from the Bill and Melinda Gates Foundation (BMFG - INV-022,816) are acknowledged. New England Biolabs provided and shipped the SARS-CoV-2 rapid colorimetric LAMP assay kit (NEB - #E2019S) free of charge. Neither BMGF nor NEB had any role in study design and/or data analysis or interpretation.

## 3 Results

### 3.1 Field evaluation in Italy

The field trial was initiated in Trieste, Italy, between November and December 2020. The local prevalence of SARS-CoV-2 positive cases over this period was 24.1% on a total of 54,770 tests. A total of 353 samples were collected in Italy from individuals referring to the ASUGI for a swab test. Of these, 313 were freshly collected, including 75 negatives, the remaining 40 were archive samples from the first wave that hit Italy in March 2020. The gender ratio between females and males of the tested individuals was 1.3 with an average age of 52 years (Table 1). The routine standard of diagnosis (SoD) RT-PCR was essentially TaqPath COVID-19 multiplex RT PC (Thermofisher) (247 samples), but other tests were occasionally used including Aptima SARS-CoV-2 Assay on Panther® System (66 samples) and in-house testing, particularly during the timeline of the first-wave peak [4]. Fresh samples were subjected to Liferiver RT-PCR and New England Biolabs (NEB) RT-LAMP less than 48 h after collection, with swabs preserved in virus transport medium (VTM) at 4 °C. Results presented in Table 1 show that Liferiver RT-PCR compared to SoD was 90% specific, with a probability that if scored negative it was actually a non-case (negative predictive value, NPV) for 84%, while sensitivity reached 96% with a probability that if scored positive it was actually a confirmed case (positive predictive value, PPV) for 97%. RT-LAMP compared with Liferiver or to SoD showed a specificity of 93% or 89%, with a PPV of 98% or 97%, while sensitivity dropped to 85% (NPV 62%) or 82% (NPV 55%), respectively. Sensitivity of 82% of LAMP compared to SoD is lower than the overall sensitivity of LAMP compared to Liferiver that was found in this study (87%, see following paragraphs), which may reflect the higher sensitivity of the technology available in Italy. Accuracy of RT LAMP compared with SoD or Liferiver was 83% (confidence interval 95%, CI, 79−87%) and 86% (95% CI, 82−90%), respectively. Concordance, determined by the Cohen's Kappa value, was 0.57 (95% CI, 0.48–0.67) for SoD and 0.66 (95% CI, 0.57–0.74) for Liferiver. These data indicate that the RT-LAMP assay, compared to either SoD or Liferiver, is specific but less sensitive than RT-PCR. However, we noted that most of the data showing discrepancies between LAMP and RT-PCR were at the upper-end of RT-PCR amplification cycles, suggesting lower viral loads. This prompted us to stratify the RT-LAMP data according to the cycle threshold (Ct) values of the Liferiver RT-PCR (Table 2A). The kit amplifies three targets, but two are enough for positivity, therefore choosing one target could miss some samples. Therefore, we opted to consider the average Ct for each sample for stratification purposes, since all targets amplified within the same range of Ct (*N* = 274, average = 31.08, cumulative standard deviation 0.7 (95% CI, 0.6–0.8)). Using the value of 30Ct, which is frequently used as discriminant between high (<30Ct) and low (≥30Ct) viral loads, sensitivity of the RT-LAMP assay <30Ct was 100%, while sensitivity dropped to 76% ≥30Ct. Further stratification into 4 steps gave the following results of sensitivity: 100% (0–25Ct), 100% (25–30Ct), 94% (30–35Ct) and 65% (35–40Ct). These data indicate that the RT-LAMP is a very good assay compared with gold-standard RT-PCR, except for samples with very low viral RNA (<8.2 × 10^3^ (95% CI, 9.6 × 10^3^ – 7.0 × 10^3^) SARS-CoV-2 genomes/mL).

**Table 1:** Parameters derived from the confusion matrix of the data divided per Country and aggregated.

	Sensitivity	Specificity	PPV	NPV	Index/Reference	*N*	Precision	Accuracy (95% CI)	*p*-value (McNemar)	Cohen's Kappa (CI 95%)	Gender ratio F/M		Average age in years (95% CI)
ITA	82%	89%	97%	55%	LAMP/SOD	353	97%	83% (79%−87%)	0.078 (4.6E-08)	0.57 (0.48–0.67)	1.3		52 (50–55)
	96%	90%	97%	84%	LifeRiver/SOD	353	97%	95% (92%−97%)	6.4E-15 (0.36)	0.84 (0.76–0.91)			
	85%	93%	98%	62%	LAMP/LifeRiver	353	98%	86% (82%−90%)	0.00015 (9.3E-08)	0.66 (0.57–0.74)			
CMR	83%	100%	100%	79%	LAMP/SOD	365	100%	90% (86%−93%)	1.1E-35 (3.3E-09)	0.8 (0.74–0.86)	0.6		38 (37–40)
ETH	98%	86%	94%	95%	LAMP/SOD	304	94%	94% (91%−97%)	6.1E-26 (0.052)	0.86 (0.80–0.93)	1.4		31 (30–33)
	99%	77%	91%	97%	LifeRiver/SOD	304	91%	92% (89%−95%)	2.6E-21 (0.00017)	0.81 (0.73–0.88)			
	95%	99%	100%	86%	LAMP/LifeRiver	304	100%	96% (93%−98%)	5.9E-21 (0.0094)	0.89 (0.84–0.95)			
KEN	82%	95%	97%	72%	LAMP/SOD	306	97%	86% (82%−90%)	5.6E-14 (1.7E-06)	0.71 (0.63–0.79)	1.5		36 (34–38)
	88%	86%	93%	78%	LifeRiver/SOD	306	93%	88% (83%−91%)	5.4E-16 (0.14)	0.72 (0.64–0.81)			
	88%	99%	99%	82%	LAMP/LifeRiver	314	99%	92% (88%−95%)	2.1E-30 (1.1E-05)	0.83 (0.77–0.9)			
NIG	72%	72%	92%	37%	LAMP/SOD	321	92%	72% (67%−77%)	1 (2.9E-09)	0.32 (0.22–0.43)	0.4	36 (35–38)	
	85%	41%	87%	38%	LifeRiver/SOD	321	87%	77% (72%−82%)	0.99 (0.64)	0.26 (0.13–0.38)			
	79%	98%	100%	54%	LAMP/LifeRiver	321	100%	83% (79%−87%)	0.11 (3.9E-12)	0.59 (0.5–0.68)			
ITA ETH KEN NIG	87%	98%	99%	70%	LAMP/LifeRiver	1292	99%	89% (87%−91%)	1.8E-37 (4.3E-25)	0.74 (0.71–0.78)			

Parameters such as: number of samples (*N*), sensitivity, specificity, negative (NPV) and positive (PPV) predictive values, precision and accuracy of the index assay, compared with the reference, were derived from the confusion matrix as described in the methods. The Cohen's Kappa coefficient was used to measure inter-rater reliability. Two different statistical tests are shown for accuracy: *p*-value and McNemar. Gender female/male ratio and average age of the tested individuals in years are also shown. Confidential interval with 95% confidence (95% CI) is also shown.

**Table 2:** Stratification of sensitivity data divided per country.

TABLE 2A										
	ITALY		ETHIOPIA		KENYA		NIGERIA		ITA-ETH-KEN—NIG	
Stratification	*N*	Sensitivity% (95% CI)	*N*	Sensitivity% (95% CI)	*N*	Sensitivity% (95% CI)	*N*	Sensitivity% (95% CI)	*N*	Sensitivity% (95% CI)
CT<25	63	100 (94–100)	95	100 (96–100)	62	98 (91–100)	41	100 (91–100)	261	99 (98–100)
CT<30	97	100 (96–100)	143	100 (97–100)	119	94 (88–98)	69	100 (95–100)	428	98 (97–99)
CT≥30	181	76 (69–82)	90	88 (79–94)	83	80 (69–88)	189	72 (65–78)	543	77 (73–81)
CT<35	167	98 (94–99)	195	99 (97–100)	175	95 (91–98)	159	95 (90–98)	696	97 (95–98)
CT≥35	111	65 (55–74)	38	74 (57–87)	27	41 (22–61)	99	55 (44–65)	275	60 (54–66)
25<CT<30	34	100 (90–100)	48	100 (93–100)	57	89 (78–96)	28	100 (88–100)	167	96 (92–99)
30<CT<35	70	94 (86–98)	52	98 (90–100)	56	98 (90–100)	90	91 (83–96)	268	95 (91–97)
35<CT<40	111	65 (55–74)	38	74 (57–87)	27	41 (22–61)	99	55 (44–65)	275	60 (54–66)

TABLE 2B	CAMEROON									
Stratification	N	Sensitivity% (95% CI)								
CT<25	129	98 (93–100)								
CT≥25	93	63 (53–73)								

A) Italy; Ethiopia; Kenya; Nigeria and aggregate data including number of samples (*N*) and sensitivity with confidence interval 95% (CI 95%).

B) Data from Cameroon compiled as above.

### 3.2 Field evaluation in sub-Saharan Africa

In parallel to the clinical trial conducted in Italy, the same protocol according to the study design was conducted in four African Countries: Cameroon, Ethiopia, Kenya and Nigeria.

### 3.3 Cameroon

The local prevalence of SARS-CoV-2 positive cases in Cameroon detected at CIRCB over the period December 2020 to February 2021 was 12.9% on a total of 6309 tests. At variance with other Countries, Cameroon opted to compare directly their SoD based on the automated Abbott RealTime SARS-CoV-2 Assay (Abbott Laboratories, USA) and not to proceed with independent QiAmp extraction and Liferiver RT-PCR in parallel. The gender ratio between females and males of the tested individuals was 0.6 with an average age of 38 years (Table 1). Specificity of RT-LAMP was 100% and sensitivity 83%, with an optimal Kappa value of 0.8 (95% CI, 0.74–0.86) (Table 1). Abbott's RealTime SARS-CoV-2 assay is a dual target RT-PCR assay for the recognition of RdRp and N genes, thus the assay cycle number (CN) output values are assay-specific and are not directly comparable to Ct values. Therefore, a different stratification was arbitrarily applied to this method: of the 129 samples that scored positive with a CN value <25, 126 were also positive in LAMP with a sensitivity of 98%, while of the 93 samples with a CN value ≥25, only 59 were positive, dropping the sensitivity to 63%, thus confirming that the sensitivity of RT-LAMP decreased when the viral RNA abundance was lower (Table 2B).

### 3.4 Ethiopia

The local prevalence of SARS-CoV-2 positive cases in Ethiopia detected at EBTI over the period of December 2020 to February 2021 was 5.8% on a total of 1890 tests. In Ethiopia the SoD RT-PCR used for routine diagnosis was from BGI Genomics. The gender ratio between females and males of the tested individuals was 1.4 with an average age of 31 years (Table 1). All the pairwise comparisons gave excellent results, with accuracy >90%, Kappa >0.8 and sensitivity exceeding 95% (Table 1). The only parameter showing a weaker behaviour was the NPV. Indeed, stratification showed a sensitivity of 100% for <30Ct and a decrease of sensitivity for samples above 30Ct, with a value of 98% between 30Ct and 35Ct and of 74% above 35Ct (Table 2A).

### 3.5 Kenya

The local prevalence of SARS-CoV-2 positive cases in Kenya detected at KEMRI over the period of December 2020 to February 2021 was 10.2% on a total of 5267 tests. In Kenya the gender ratio between females and males was 1.5 with an average age of 36 years (Table 1). In this country, the SoD RT-PCR used for routine diagnosis was from various sources: DaAn Novel Coronavirus detection kit (DaAn gene Co. China), SD Biosensor nCOV real time detection kit (SD Biosensor Inc. Korea), BGI Real-Time Fluorescent RT-PCR kit (BGI, China), AccuPower SARS-CoV-2 RT-PCR kit (Bioneer, South Korea). Such variability affects the comparsion of SoD with Liferiver or LAMP (Table 1). Nonetheless, comparison of RT LAMP with Liferiver RT-PCR remained in line with the other Countries, while stratification showed a sensitivity of 94% for samples <30Ct and of 80% for ≥30Ct (Tables 1 and 2A).

### 3.6 Nigeria

The local prevalence of SARS-CoV-2 positive cases in Nigeria detected in Maiduguri over the period of December 2020-March 2021 was 10.8% on a total of 5859 tests. In Nigeria the gender ratio between females and males of the tested individuals was 0.4 with an average age of 36 years (Table 1). Results showed that comparisons with SoD gave very poor parameters, with sensitivity dropping to 85% comparing Liferiver to SoD, or even 72% comparing LAMP with SoD. This could be explained by the fact that availability of reagents, as already shown for Kenya, was entirely dependent on donations from various sources routed through the NCDC. Indeed, specificity/PPV reached 98/100% when comparing LAMP with Liferiver RT-PCR, with a sensitivity of 79%, NPV of 54%, accuracy of 83% and *K* = 0.59 (Table 1). Hence, results from standardized protocols for RNA extraction, RT-PCR and RT-LAMP were in line with the other Countries, while SoD suffered from poor performance, emphasising the importance of better and more standardized assays for routine analysis. Stratification of LAMP/Liferiver data showed sensitivity of 100% for samples <30Ct, reduced to 91% for 30–35Ct and 55% for 35–40Ct (Table 2A).

### 3.7 Overall performance

**Fig. 1:**
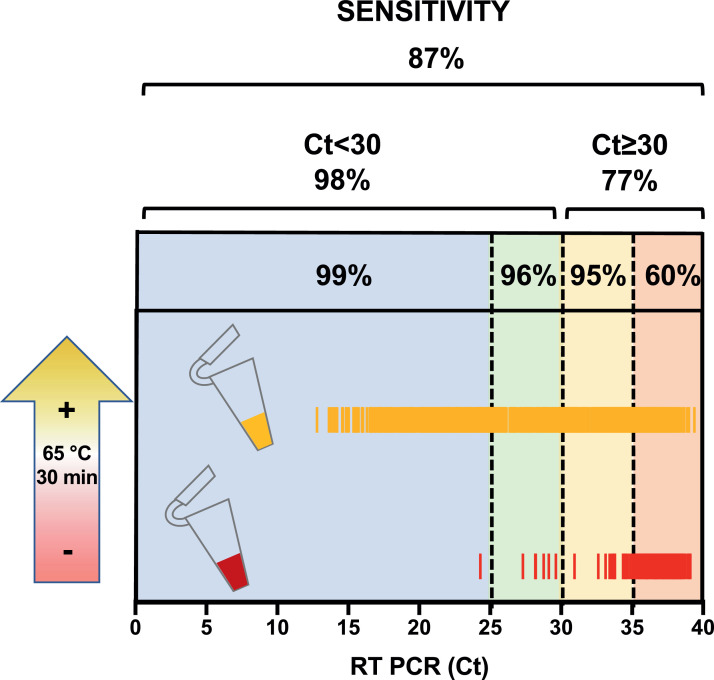
Stratification of sensitivities on RT-PCR Ct values. Stratification of the RT LAMP colorimetric results (positive/negative) on the RT-PCR (Liferiver) Ct values. The RT LAMP is a naked-eye colorimetric assay where the reaction incubated at 65 °C for 30 min turns from red (negative) to yellow (positive). Aggregated data from Italy, Ethiopia, Kenya and Nigeria are shown as yellow (positive) and red (negative) bars. Data for sensitivity within the various stratification windows are described in Table 2. To better correlate Ct values with SARS-CoV-2 RNA genomes a standard curve was build and used to interpolate values from RT-PCR. With this method 25 Ct correspond to 5.9 × 10^6^ (95% CI, 6.8 × 10^6^ – 5.2 × 10^6^) SARS-CoV-2 genomes/mL; 30 Ct correspond to 2.2 × 10^5^ (95% CI, 2.4 × 10^5^ – 1.8 × 10^5^) genomes/mL; 35 Ct correspond to 8.2 × 10^3^ (95% CI, 9.6 × 10^3^ – 7.0 × 10^3^) genomes/mL. (For interpretation of the references to color in this figure legend, the reader is referred to the web version of this article).

Overall comparison of RT LAMP with Liferiver RT-PCR in the Countries where this was possible (all except Cameroon) showed an accuracy of 89% (95% CI, 87−91%) with very good specificity (98%) and PPV (99%), while sensitivity was 87% with a NPV of 70%, but increased to 98% below 30Ct (viral load >2.2 × 10^5^ (95% CI, 2.4 × 10^5^ – 1.8 × 10^5^) SARS-CoV-2 genomes/mL) with NPV of 98%, and to 97% for samples <35Ct (viral load >8.2 × 10^3^ (95% CI, 9.6 × 10^3^ – 7.0 × 10^3^) SARS-CoV-2 genomes/mL) with NPV of 94% (Table 1 and 2A). Above 30Ct, the sensitivity gradually decreased to 95% for 30–35Ct and 60% for 35–40Ct (Fig. 1).

## 4 Discussion

The current SARS-CoV-2 circulation in Africa is worrying, with heavy impact on the economies and having critical social and economic implications. The number of newly detected COVID-19 cases in Ethiopia continues to increase rapidly, with more than 25% positivity rate [23]. Similarly, Cameroon is still experiencing a growth of cases, while in other countries, such as Kenya and Nigeria, the situation is more stable. Italy was the first European country to be hit by the SARS-CoV-2 pandemic wave in February 2020, it experienced a second wave in the autumn, and is currently undergoing a third wave [24]. While vaccine rollout is in progress worldwide, its distribution is uneven, with marked differences in the pace of inoculation. Hence, continuous surveillance for SARS-CoV-2 will continue to be a priority until sufficient coverage is obtained.

Affordable diagnostics for COVID-19 can have positive economic and social impacts in several ways, particularly by improving care, enabling tracking and contact tracing, and informing interventions such as lockdowns, prevention of transmission by cluster detection, and predicting the viral spread at community level. They also allow evidence-guided “return-to-normal”, which is essential to revitalize local economies. Although the pandemic has resulted in increased funding for diagnostic research and development, this has been overwhelmingly focused in high-income Countries. For this reason, access to diagnostics in LMIC/LDCs has recently received attention from many intergovernmental agencies. Any attempt in this direction has to consider the evolution of various diagnostic technologies [5,25]. For example, during the early stages of the pandemic, tests such as the RT-PCR were predominantly used for diagnosis and still represent the gold-standard for SARS-CoV-2 detection [2]. However, these tests are generally expensive, even for high-income Countries, and their application in LMIC/LDCs can be challenging and further drain already-stressed health systems. For this reason, there is an increasing focus on tests that are affordable, rapid, and can be applied frequently for repeated routine testing.

RT-LAMP has received a lot of attention as a cost-effective molecular test for the detection of SARS-CoV-2 [26,27]. Our clinical data confirm that the RT-LAMP assay has a high specificity (98%) and PPV (99%), and would be suitable for identifying individuals with a medium-high SARS-CoV-2 viral load (sensitivity 98%, Ct<30, viral load >2.2 × 10^5^ genomes/mL) with NPV of 98%, and to 97% for samples <35Ct (viral load >8.2 × 10^3^ genomes/mL)), while for lower viral loads the sensitivity of the RT-LAMP assay gradually decreases, remaining acceptable up to <35Ct (97%), and dropping to 60%>35Ct (Table 2). These results are in line with those of Dao Thi et al. [27] who demonstrated a sensitivity of 97,5% and a specificity of 99,7% with a limited number of surplus pharyngeal samples collected in Germany. Compared to the Isopollo COVID-19 detection kit (M Monitor, South Korea), with a sensitivity of 61.9%, the performance was certainly better [28]. Automated commercial platforms based on RT-LAMP reported comparable results with a decrease of sensitivity at lower viral loads, although differences in operational processing of samples may influence the comparison [29].

Although there are no doubts on the Ct values that are considered positive in RT-PCR for SARS-CoV-2 detection, there is still some debate on which Ct value, or better viral load, for a positive RT-PCR result should be considered relevant for transmission. According to the kit specifications and our analysis, the analytical sensitivity of the Liferiver RT-PCR, used in our study, is 10^3^ copies/mL using the QIAmp RNA extraction method; which aligns with the values obtained for the majority of amplification methods [15,16,30,31]. Long-term shedders of SARS-CoV-2 could experience higher and lower viral loads depending on the day of collection, but the general trend is that higher viral loads are required for isolation, although infectivity cannot be totally ruled out based on Vero cells’ infection only [32,33]. From our experience and from a meta-analysis, the chances of culturing SARS-CoV-2 from swabs when the RT-PCR Ct value of the specimen is >25 is very low (unpublished observations) [34]. More systematic studies reported that culturing virus declined to 8% in specimens with Ct >35 [35]. Another study analysed positive specimens with known Ct values and found that 70% could be cultured if they had a Ct ≤ 25, but less than 3% with a Ct >35 [36]. Similarly, others reported that with Ct >24 they were not able to culture the virus [37]. It may be noted also that SARS-CoV-2 can still be detected for weeks after clearance of symptoms using RT-PCR, with no cell culture infectivity [38,39]. Therefore, we can conclude that the colorimetric RT-LAMP assay is able to detect viral RNA in samples from individuals that can transmit the virus, but it may miss those that do not transmit efficiently at later stages of the diseases, or individuals at the very onset of illness, when the viral load is very low. The latter is certainly a limitation of the colorimetric RT-LAMP described here, which needs to be carefully considered when implementing its use for diagnostic or surveillance. Another limitation of RT-LAMP is that it requires careful optimization of a number of primers and is not quantitative because the amplification of concatamers is not linear as it is in the RT-PCR [40]. However, these limitations should be weighed against the many advantages. LAMP is a molecular assay that is growing in use as a cost-effective tool for SARS-CoV-2 surveillance and diagnostics [26,[41], [42], [43]]. Not only it is valid as a naked-eye colorimetric assay, as herein demonstrated, but it is also amenable to spectrophotometric quantification [27]. Furthermore, point-of-care (POC) testing based on RT LAMP for SARS-CoV-2 detection is being developed for deployment in remote areas [44,45]. Interestingly, various one-tube applications for SARS-CoV-2 RT-LAMP have been proposed [43], including pre-print studies (doi:10.31224/osf.io/ed85s; doi:10.1101/2020.04.21.052530).

In this work, we provide evidence supporting the deployment of RT-LAMP in LMIC/LDCs as alternative to RT-PCR. Another alternative to molecular assays in these Countries is represented by immunochromatographic assays based on rapid antigen testing. However, while specificity is generally high, sensitivity is quite variable between different tests [46]. A recent meta-analysis on 19 clinical studies showed a reasonably good specificity, ranging from 92.4 to 100%, while sensitivity varied greatly, between 28.9 and 98.3% [47]. Therefore, such rapid tests can be useful for large-scale screening, but cannot generally substitute for molecular tests, including RT LAMP, except perhaps those few with the best performances.

Africa is a continent that pays an immense toll to infectious disease, owing to several factors, including poor surveillance capability. Potential solutions for cost-effective diagnostics of infectious disease require rigorous clinical field-testing in SSA countries, in order to overcome daily challenges that are encountered locally. In this work, we provide convincing clinical evidence of the validity of the RT-LAMP approach in detecting SARS-CoV-2 in SSA, alongside a framework for future study design.

## Authors’ contributions

MMB, MB, JF, EAL, EB, SZ and AM contributed to the conception, design, acquisition and analysis of data, writing, reviewing and editing. LDC, PM and AM contributed to statistical analysis of data. All other authors contribute to the acquisition of data and local clinical and experimental management of the project.

## Funding

This work was supported, in whole or in part, by the Bill & Melinda Gates Foundation [Grant No. INV-022,816]. Under the grant conditions of the Foundation, a Creative Commons Attribution 4.0 Generic License has already been assigned to the Author Accepted Manuscript version that might arise from this submission.

## Data sharing statement

Data used in this study are available from the corresponding authors upon reasonable request.

## Declaration of Competing Interest

The authors declare that this study was supported by the Bill & Melinda Gates Foundation [Grant Number INV-022,816]. Furthermore, New England Biolabs provided reagents free of charge. The authors have no additional interests to declare.
